# Kinematics Error Compensation for a Surface Measurement Probe on an Ultra-Precision Turning Machine

**DOI:** 10.3390/mi9070334

**Published:** 2018-07-02

**Authors:** Duo Li, Xiangqian Jiang, Zhen Tong, Liam Blunt

**Affiliations:** Engineering and Physical Sciences Research Council (EPSRC) Future Metrology Hub, University of Huddersfield, Queensgate, Huddersfield HD1 3DH, UK; zhen.tong@hud.ac.uk (Z.T.); l.a.blunt@hud.ac.uk (L.B.)

**Keywords:** on-machine surface measurement, kinematics error model, error measurement, error compensation, ultra-precision machine tool

## Abstract

In order to enhance the measurement availability for manufacturing applications, on-machine surface measurement (OMSM) is integrated onto the machine tools, which avoids the errors caused by re-positioning workpieces and utilizes the machine axes to extend the measuring range as well. However, due to the fact that measurement probe actuation is performed using the machine tool axes, the inherent kinematics error will inevitably induce additional deviations onto the OMSM results. This paper presents a systematic methodology of kinematics error modelling, measurement, and compensation for OMSM on an ultra-precision turning lathe. According to the measurement task, a selective kinematics error model is established with four primary error components in the sensitive measurement direction, based on multi-body theory and a homogeneous transformation matrix (HTM). In order to separate the artefact error from the measurement results, the selected error components are measured using the reversal method. The measured error value agrees well with the machine tool’s specification and a kinematics error map is generated for further compensation. To verify the effectiveness of the proposed kinematics error modelling, measurement, and compensation, an OMSM experiment of an optically flat mirror is carried out. The result indicates the OMSM is the superposition of the sample surface form error and the machine tool kinematics error. With the implementation of compensation, the accuracy of the characterized flatness error from the OMSM improves by 67%.

## 1. Introduction

Ultra-precision manufacturing has developed over the decades to produce highly demanding surfaces for optical, electronic, or mechanical applications [1]. In order to enhance the availability of metrology for such applications, there has been a shift in the metrology approach from offline, lab-based solutions towards the use of metrology within manufacturing platforms [2]. The advantage of on-machine surface measurement (OMSM) is that the coordinate system between the machining and measurement processes is kept consistent, which avoids the errors caused by re-positioning workpieces. Moreover, the machine axes are utilized to extend the measuring range [3]. However, due to the fact that measurement probe actuation is performed on the machine, the machine tool kinematics error will inevitably induce additional errors to OMSM results. Therefore, it is necessary to model, measure, and compensate the machine tool kinematics errors for OMSM in order to improve the measurement accuracy.

Modelling and compensation of kinematics errors for ultra-precision machine tools have received much interest [4,5,6,7]. Kong et al. [8] developed a kinematics error model for two-axis ultra-precision turning machines. Software error compensation was carried out by the modification of the ideal tool path in the numerical control program. Flat and tapered machining experiments have been conducted to verify the established theoretical kinematics model and higher surface accuracy was achieved with the implementation of the proposed compensation method. Chen et al. [9] studied the modelling of volumetric error and its sensitivity analysis for a five-axis ultra-precision machine. The volumetric error model, including 37 error components, was established based on rigid body kinematics and a homogeneous transformation matrix. In addition a sensitivity analysis was carried out for the purposes of accuracy design and manufacture specification of the machine tool. Yu et al. [10] analysed the main sources of machining errors and their effect on diamond-turned micro-structured surfaces. Sliding error, as well as the dynamic error of the fast tool servo mechanism, were identified and compensated for by means of tool path modification. Machining experiments of typical micro-structured surfaces demonstrated the effect of the component errors on profile accuracy and verified the effectiveness of the proposed compensation methods. Gao et al. [11] studied the measurement and compensation of error motions of a diamond turning machine for nano-fabrication of large sinusoidal metrology grids. The out-of-straightness of the X slide, and the axial and angular motion of the spindle, were measured, respectively. The experiment results indicated that the out-of-flatness of the workpiece was reduced from 0.27 to 0.12 μm after compensation machining by utilizing a fast tool servo unit. Liu et al. [12] classified the machining errors into five categories for a three-axis turning machine according to the coordinate distortions direction. The author pointed out that the effect of kinematic errors on the coordinate distortion and form accuracy are dependent on the surface type. A one plane-spherical surface was fabricated to identify the main machining errors on an ultra-precision turning machine. Both simulation and machining experiments were implemented to verify the effectiveness of the kinematic error model and compensation strategy. Most previous work focused on machine tool error modelling for the improvement of machining accuracy. There is relatively little research on the comprehensive investigation of kinematics error modelling, measurement, and compensation for on-machine surface measurement applications. To address this issue, this paper will firstly develop a selective kinematics error model according to the measurement task and the configuration of the machine tool. The measurement processes for selective kinematics errors in the sensitive direction are also presented. Next, a kinematics error map for OMSM compensation is generated using the established error model and error measurement results. Finally, an OMSM experiment of an optically flat mirror is carried out to verify the effectiveness of the proposed modelling, measurement, and compensation. 

## 2. Overview of OMSM System

**Figure 1:**
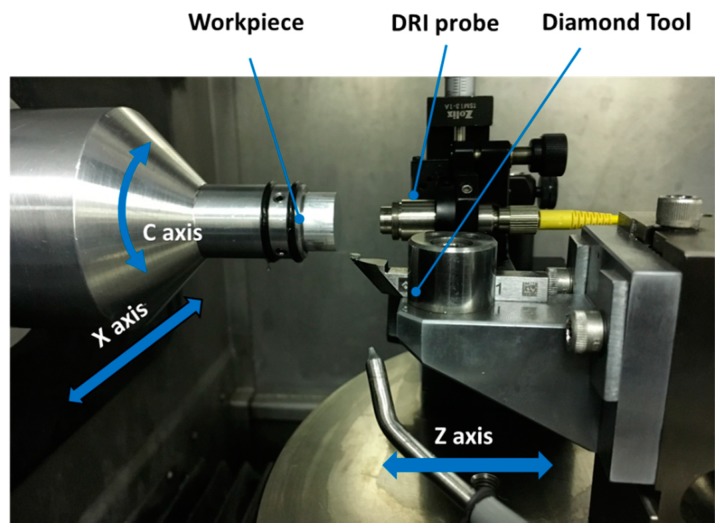
On-machine surface measurement system configuration.

In this work, an optical interferometric probe, as the on-machine surface measurement instrument, is integrated onto a three-axis ultra-precision turning lathe (Nanoform 250 Ametek Precitech, Keene, NH, USA), equipped with two linear hydrostatic axes and one rotational air bearing spindle. The probe, termed dispersed reference interferometry (DRI) [13], works on the principle of a modified Michelson interferometer with chromatic dispersion purposefully added in the reference arm, resulting in a wavelength-dependent optical path length. The dynamic design enables its measuring capacity to nanometre resolution (0.6 nm) and millimetre vertical range (800 µm). The use of a low coherence light source lends itself to an optical fibre-based implementation, giving the potential for remote configuration and miniaturization in the manufacturing environment. The DRI probe is mounted beside the diamond tool holder on the Z slide and aligned coaxially to the spindle axis before OMSM operations. The system configuration is illustrated in Figure 1.

**Figure 2:**
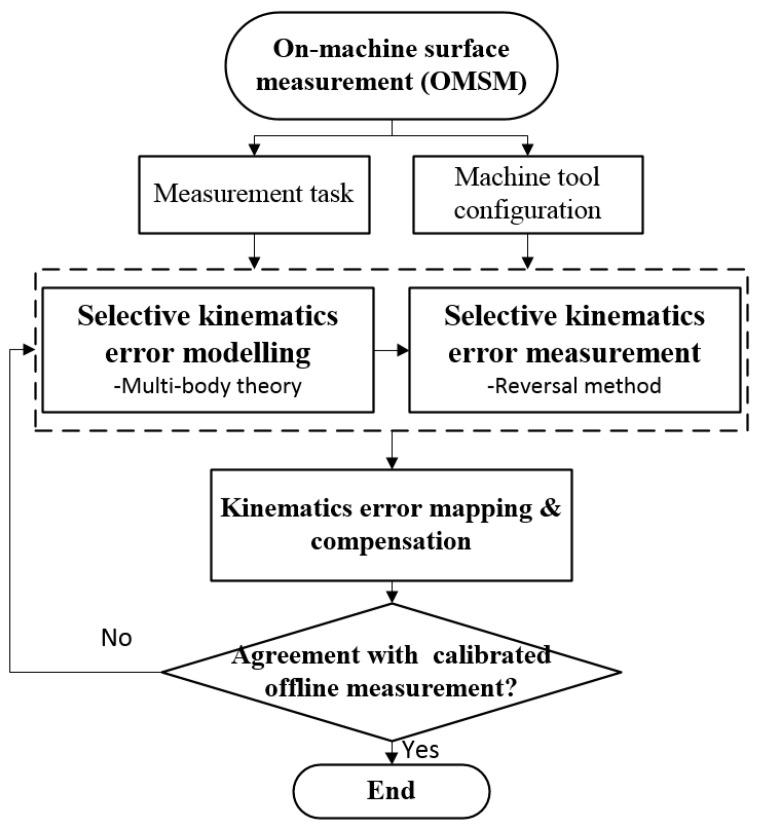
Flowchart of the kinematics error compensation for on-machine surface measurement (OMSM).

For on-machine measurement, the DRI probe is carried by the machine tool axes to scan over the sample surface. However, due to the inherent mechanical imperfections, wear of the machine tool elements and stage misalignments [14], and deviation from the programmed scanning path, will induce undesirable errors in the measurement results. Hence, the machine tool kinematics error needs to be modelled, measured, and compensated for OMSM results. The flowchart of the proposed methodology is illustrated in Figure 2. According to the measurement task and machine tool configuration, a selective kinematics error modelling and measurement process will be carried out. The machine tool kinematics error in the scanning region is consequently mapped in order to compensate for the OMSM result. To validate the proposed methodology, the OMSM result is compared with offline measurement results. 

## 3. Kinematics Error Modelling

**Figure 3:**
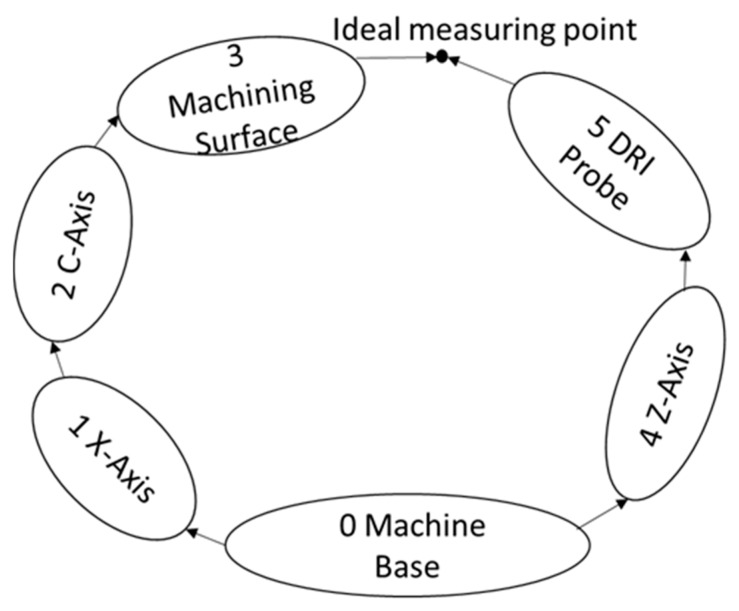
Kinematics error chain for an on-machine surface measurement system.

Kinematics error modelling for multi-axis machine tools is based on the multi-body system theory [15]. The multi-body system theory offers a comprehensive description of general mechanical systems utilizing the lower-order body topological structure. Using a homogeneous transformation matrix (HTM), the relationship between multi-coordinates can be established and spatially-distributed single error components are consequently synthesized as a volumetric error model. For the machine tool configuration in the current work, there are two kinematics error chains, as illustrated in Figure 3. One is from the machine base to the machining surface, and the other is from the machine base to the DRI probe. 

**Figure 4:**
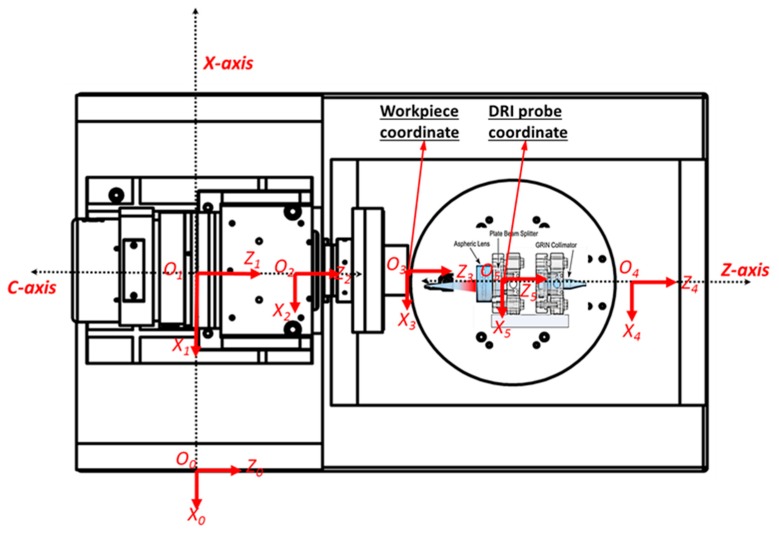
Configuration of the machine tool coordinate systems.

The overall configuration of the machine tool coordinate systems is shown in Figure 4. The spatial relationship between adjacent coordinate systems can be mathematically described using the homogeneous transformation matrix. For example, the transformation matrix Tji describes the coordinate transformation from coordinate system *j* to coordinate system *i*. By sequential multiplication of four transformation terms, the comprehensive transformation matrix Tji between two adjacent bodies can be formulated as:

(1) Tji=TjilocTjiloceTjimTjime

where Tjiloc is the location transformation matrix, Tjiloce is the location error transformation matrix, Tjim is the motion (translation or rotation) transformation matrix, and Tjime is the motion (translation or rotation) error transformation matrix. According to the kinematics chain structure illustrated in Figure 3, the comprehensive transformation matrices between adjacent coordinates can, thus, be derived and listed in the following Table 1.

By transferring to a common machine base coordinate system from the two chains, we have:

(2) T=∏Tji

(3) T30=T10T21T32

(4) T50=T40T54

The volumetric error vector, which describes the relative displacement deviation between the DRI probe and the workpiece surface can, thus, be derived as:

(5) [ExEyEz1]=T30[p3xp3yp3z1]−T50[p5xp5yp5z1]

All the error variables presented above follow the convention according to the ISO 230-1 [16]. For three-axis turning machines there are 21 error components in the established kinematics model, including 18 error terms for individual axes (6 × 3) and three error terms describing the relative location between the three axes. However, it is time-consuming and unnecessary to model and measure all the error components. More attention should be paid to the primary error components in the sensitive direction because they directly influence the surface measurement accuracy. In the current work, the Z direction illustrated in Figure 1 is considered as the measurement-sensitive direction according to the measurement task. Four error components are selected as primary factors affecting the on-machine measurement results in the sensitive direction. They are, respectively, the X axis straightness in the Z direction, *E_ZX_*; the squareness error between the X and C axes, *E_BOC_*; the C axis axial error, *E_ZC_*; and the C axis tilt error, *E_BC_*. These four error components will be measured, synthesized, and employed to generate the kinematic error map in Section 4. Furthermore, the selective kinematics error model in the Z direction can be simplified as follows:

(6) $$E_{Z}=z-E_{ZC}-E_{ZX}+x\;E_{BC}+x\;E_{BOC}$$

**Figure 5:**
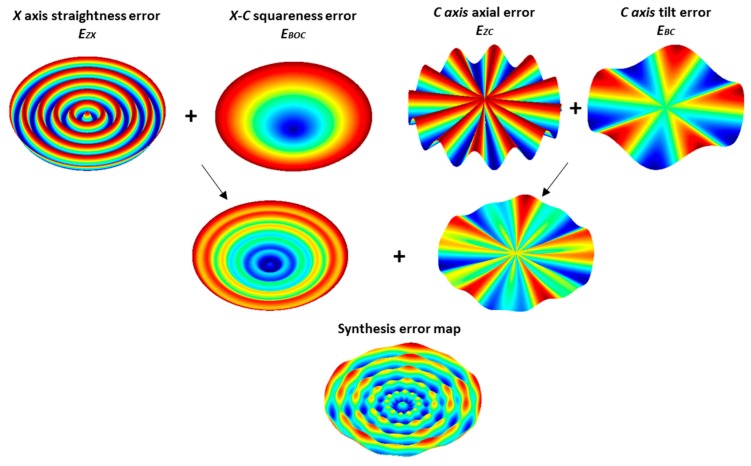
Simulation of the kinematics error effect on the OMSM results.

From the formula above, it can be seen that the synthesized error in the Z direction comprises the four selective error components. With the derived selective kinematics error model, the individual and combined effect of these errors on OMSM results are numerically simulated and illustrated as 3D error maps in Figure 5. The X axis straightness error in the Z direction *E_ZX_* will cause the wavy pattern along the radial direction, while the squareness error *E_BOC_* between the C and X axes in the X-Z plane results in the cone-shaped surface. The C axis motion errors, including axial motion *E_ZC_* and tilt error *E_BC_*, will induce several circumferential ripples, whose number depends on the spindle motion error characteristics. It can also be inferred that the squareness error and C axis tilt error tends to exaggerate the motion error in the Z direction with increasing sample radius. Compensation of the error components *E_BOC_* and *E_BC_* should receive more attention for the on-machine measurement of large-scale surfaces.

## 4. Kinematics Error Measurement

Ultra-precision machine tools are equipped with high-precision linear hydrostatic guideways and air-bearing spindles. The motion errors of the linear and rotational axes often lie in the sub-micrometre, even in the nanometer, range [17,18,19]. Without the influence of the inherent surface form error on the artefact, error separation techniques have been widely adopted for precision measurement of error motions on ultra-precision machine tools [20,21,22]. Among them, the reversal method is considered simple and accurate for the measurement of part features without reference to an externally-calibrated artefact [23]. This section proposes a simple scheme for machine tool kinematics error measurement at the nanometric level, with capacitance probes (Lion Precision C8, Minnesota, MN, USA) and flat artefacts. The maximum sampling frequency of the capacitance probes used is up to 1 kHz and the displacement measurement resolution is 0.08 nm. The testing of the capacitance probe does not indicate drifting issues, which is included in Appendix A. Furthermore, the 2 mm spot size also automatically filters out short wavelength errors on the target surface so that the artefact surface finish will not affect the measurement. In the following part, the measurement process for the X axis straightness in the Z direction *E_ZX_*, C axis axial error *E_ZC_*, C axis tilt error *E_BC_*, and squareness error between X and C axes’ *E_BOC_* will be respectively described. 

### 4.1. X Axis Straightness Error

**Figure 6:**
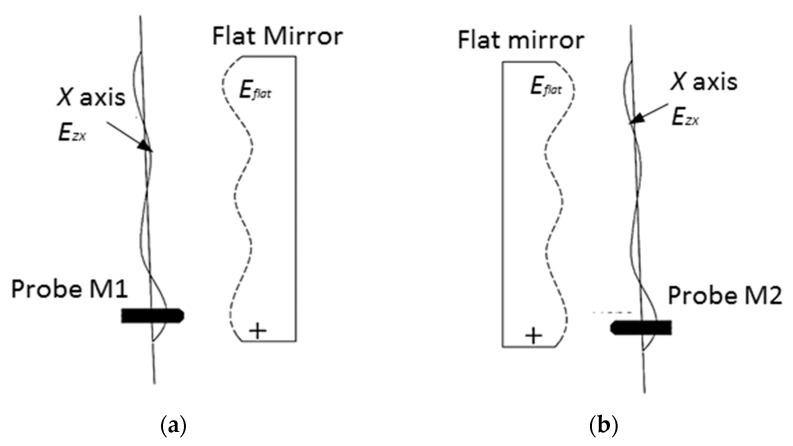
Schematic diagram of the *E_ZX_* measurement. (**a**) Before reversal operation, (**b**) After reversal operation.

**Figure 7:**
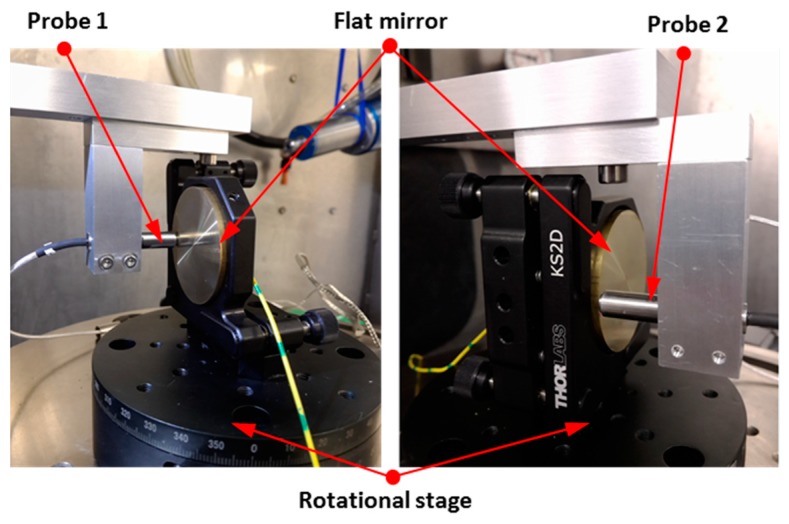
Experimental setup of the *E_ZX_* measurement using the reversal method.

The schematic diagram and experimental setup of the *E_ZX_* measurement using the reversal method are respectively shown in Figure 6 and Figure 7. A metal flat mirror was mounted on the Z axis stage and kept stationary. The capacitance probe was carried on the X slide and scanned over the mirror for 38 mm at 10 mm/min. Subsequently, the mirror was rotated 180° using a manual rotational stage and the mirror was scanned again after the reversal operation. The forward and reversal scanning were carried out three times and the averaged output was used for error separation.

The two measurements are respectively denoted as *M*_1_ and *M*_2_. According to the reversal principle, the straightness error *E_ZX_* can be separated from the surface error of the flat mirror *E_flat_* and calculated by:

(7) $$\left\{ \begin{array}{l} M_{1}=E_{flat}-E_{ZX}\\ M_{2}=E_{flat}+E_{ZX} \end{array}\right.$$

(8) $$E_{ZX}=\frac{1}{2}(M_{2}-M_{1})$$

**Figure 8:**
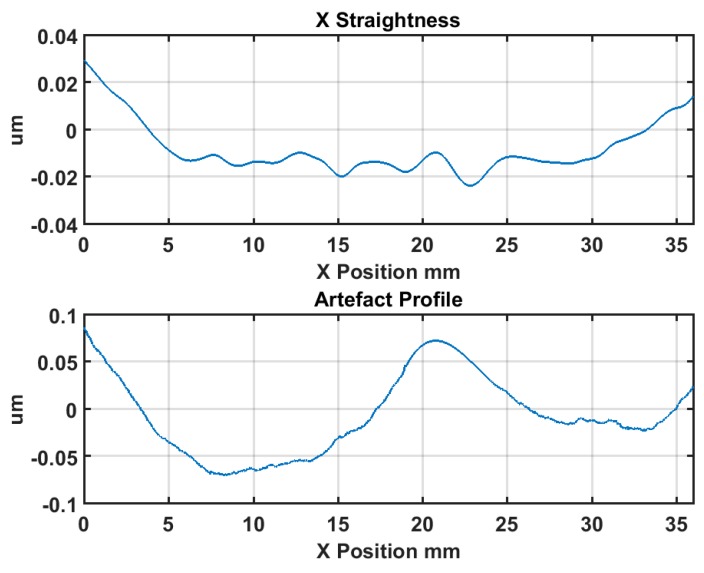
Error separation results of straightness error *E_ZX_* and artefact profile error *E_flat_*.

The error separation results are shown in Figure 8. As shown in the upper plot, the straightness error of the X axis *E_ZX_* is 52.6 nm over a 38 mm measurement range, in accordance with the machine tool specification (50 nm over 25 mm range). It should be noted that the measuring probe was set up on the Z axis at the same height as the spindle axis. Thus, the Abbé errors would be included in the measurement results. In fact, under such circumstances, the measured and separated errors (using the reversal method) includes the pure X axis straightness in its carriage axis line and the Abbé errors. However, it is not an issue for OMSM compensation in this work. As in the OMSM process, the DRI probe was also set up on the Z axis at the same height with the spindle axis. Therefore, this combined error can be compensated directly.

### 4.2. C Axis Axial and Tilt Error

**Figure 9:**
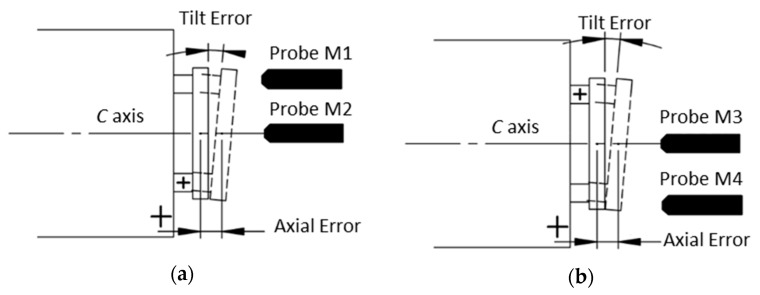
Schematic diagram of the facial reversal method. (**a**) Before reversal operation, (**b**) After reversal operation.

**Figure 10:**
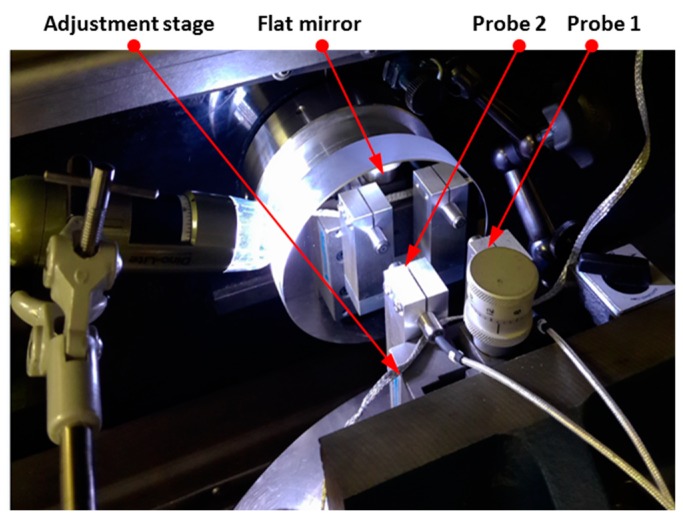
Experiment setup of the facial reversal measurement.

For C axis error measurement, the facial reversal method is utilized to measure the axial and tilt motion error [24]. Facial error motion, which is parallel to the rotational axis, is the superposition of the axial error and the tilt error. The schematic diagram and experimental setup of the facial reversal measurement is, respectively, illustrated in Figure 9 and Figure 10. Two capacitance probes were set separately at the distance *L*. After the forward measurement (output *M*_1_ and *M*_2_), the flat mirror was rotated 180° relative to the C axis and the two probes were moved according to Figure 9b. Next, the reversal measurement (output *M*_3_ and *M*_4_) was performed. The rotational speed was set as 20 revolutions per minute for forward and reversal measurements, and the measurement was performed over 20 revolutions. It is noted that the measurement outputs *M*_1_ and *M*_4_ are the combination of the flat form error *E_flat_*, the tilt error *E_BC_*, and the axial error *E_ZC_*. 

According to the facial reversal principle, the form error *E_flat_*, the tilt error *E_BC_*, and the axial error *E_ZC_* can be separated by:

(9) $$\left\{ \begin{array}{l} M_{1}=E_{Flat}+L\times E_{BC}+E_{ZC}\\ M_{4}=E_{Flat}-L\times E_{BC}+E_{ZC}\\ M_{2}=M_{3}=E_{ZC} \end{array}\right.$$

(10) $$\left\{ \begin{array}{l} E_{ZC}=\frac{\left(M_{2}+M_{3}\right)}{2}\\ E_{BC}=\frac{\left(M_{1}-M_{4}\right)}{2L}\\ E_{Flat}=\frac{\left(M_{1}+M_{4}\right)}{2}-E_{ZC} \end{array}\right.$$

**Figure 11:**
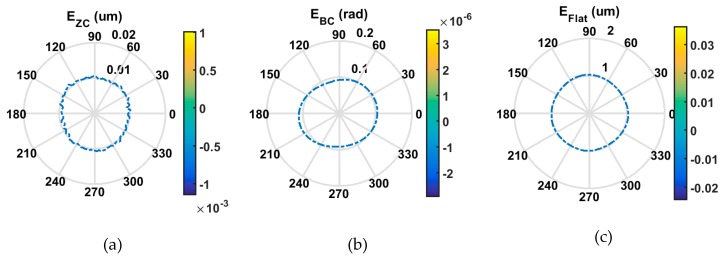
Error separation of *C* axis axial error *E_ZC_*, tilt error *E_BC_*, and artefact profile error *E_flat_*. (**a**) Axial error *E_ZC_*, (**b**) Tilt error *E_BC_*, (**c**) Artefact profile error *E_flat_*.

Figure 11 illustrates the error separation results of the *C* axis measurement. Axial error *E_ZC_* is measured to be 4.4 nm, which is within the range of the machine tool specification (less than 15 nm). It is noticed that the tilt error *E_BC_* shows a two-lobe pattern, as shown in the polar plot (Figure 11b).

### 4.3. Squareness Error between X and C Axes

**Figure 12:**
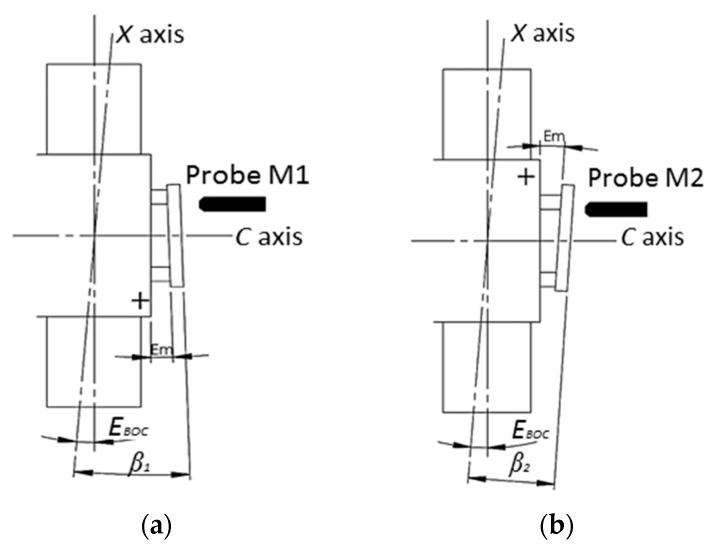
Schematic diagram of *E_BOC_* measurement. (**a**) Before reversal operation, (**b**) After reversal operation.

**Figure 13:**
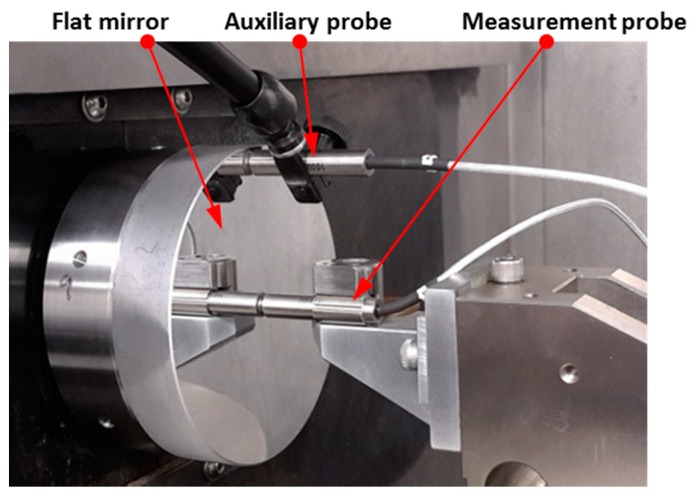
Experimental setup of *E_BOC_* measurement.

The squareness error *E_BOC_* tends to induce a linear trend deviation on the surface measurement results. The schematic diagram and experimental setup of the squareness error *E_BOC_* measurement are respectively illustrated in Figure 12 and Figure 13. The same flat mirror was mounted on the *C* axis and the measurement using a capacitance probe was performed by X directional scanning over the mirror surface. Linear slope *β* can be calculated by linear fitting of the acquisition data, which describes the angle between the linear X axis motion and the flat mirror, as illustrated in Figure 12. Then, the *C* axis was rotated 180° and the scanning along the X axis was performed again. The forward and reversal measurement were carried out three times and the averaged outputs were used for the squareness error calculation. 

**Figure 14:**
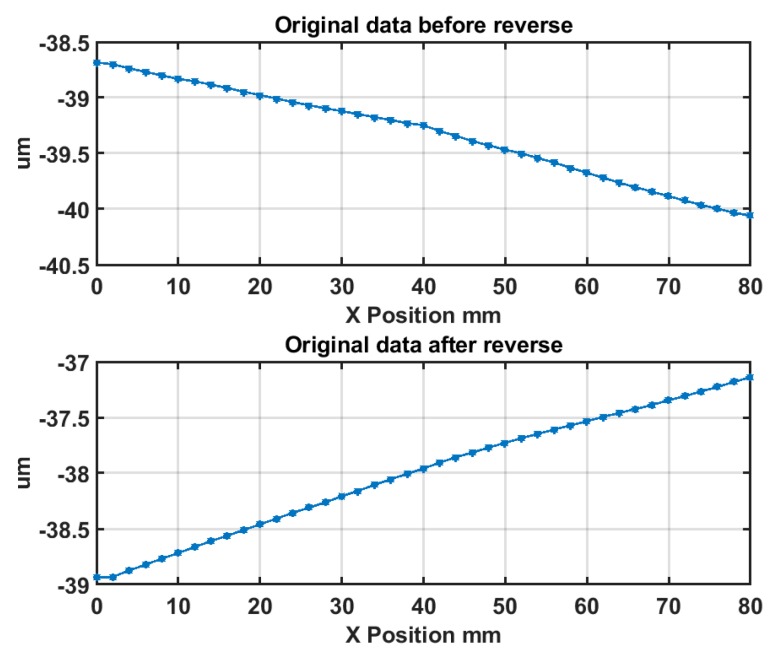
Squareness error *E_BOC_* measurement result.

The averaged measurement results are shown in Figure 14. The squareness error between the X axis and the C axis *E_BOC_* can be derived by:

(11) $$E_{BOC}=\frac{1}{2}\times \left(\beta_{1}+\beta_{2}\right)-E_{tilt}$$

where *β*_1_ and *β*_2_ are the fitted angles derived respectively from the fitting of the two measurement data sets. The squareness error *E_BOC_* is calculated to be 0.08 arc sec.

### 4.4. Kinematics Error Map Generation

**Figure 15:**
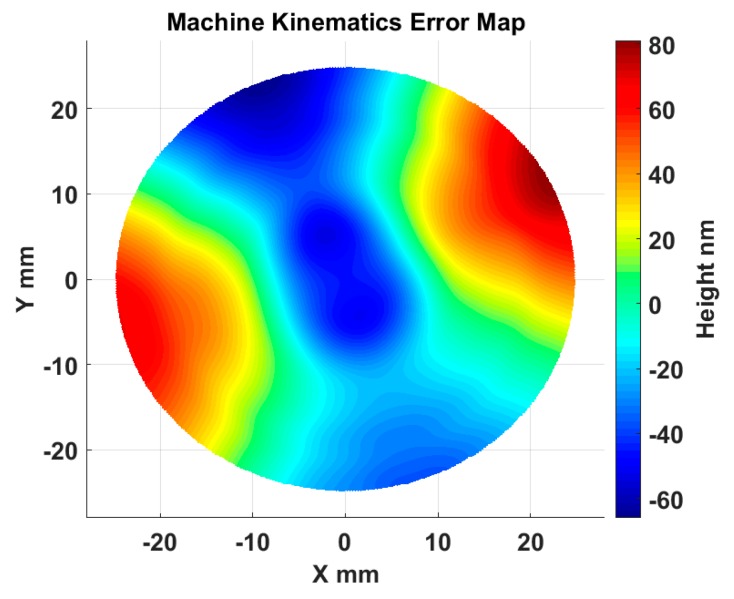
Machine tool kinematics error map.

Based on the established kinematics error model and error measurement results presented above, the machine tool kinematics error was numerically mapped. The kinematics error can be stored as a look-up table for further compensation of on-machine measurement results. It can be observed in Figure 15 that the kinematics error map is dominated by a 2 upr (undulations per revolution) component along the circumferential direction, which mainly results from the *C* axis tilt error motion *E_BC_*, corresponding to the measurement result shown in Figure 11b.

## 5. OMSM Experiment and Analysis

**Figure 16:**
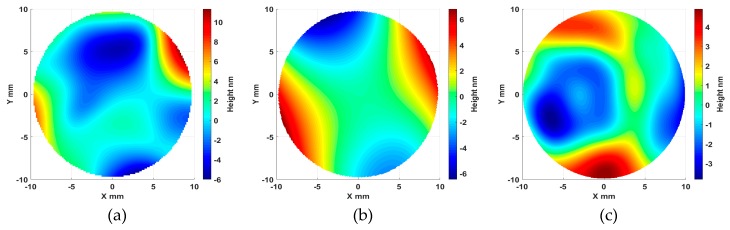
Dispersed reference interferometry (DRI) on-machine measurement (**a**), kinematics error map (**b**), and Fisba measurement of the optically flat mirror (**c**).

In order to verify the proposed kinematics error modelling, measurement, and compensation, an OMSM experiment of an optically flat mirror (Edmund Optics, Barrington, NJ, USA) was carried out. The optical mirror used in the experiment was a superpolished surface. In this way, we can better investigate the effect of the machine kinematics error on the OMSM form measurement without other factors’ effects, such as turning marks, burrs, and defects. The use of a flat surface in the experiment was also intended to minimize the effect of linearity errors from the DRI probe on the measurement results. The mirror was mounted on the spindle chuck and scanned by the DRI probe in a spiral path (the measurement radius was 10 mm). For comparison, the flat mirror was also measured offline by a calibrated Twyman–Green interferometer (Fisba FS10, FISBA AG, St. Gallen, Switzerland). The offline result was regarded as an accurate representation of the flat surface form. On-machine and offline measurements were performed three times to evaluate the repeatability. The results of the DRI measurement, kinematics error map, and Fisba measurement are respectively shown in Figure 16. The kinematics error used in this experiment was interpolated from the modelled kinematics error map shown in Figure 15.

**Figure 17:**
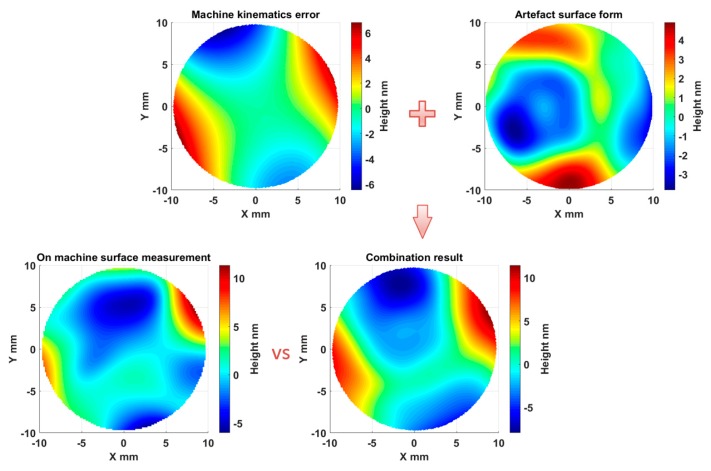
DRI on-machine measurement vs. the combination of the kinematics error and Fisba measurement.

By means of combination of the kinematics error map and the Fisba measurement, the results in Figure 17 indicate that the DRI on-machine surface measurement result of the optically flat mirror agrees with the superposition of the machine tool kinematics error and the sample form error. With the aid of kinematics error mapping established above, the on-machine probing data was compensated by subtracting the kinematics error. The characterized flatness from the DRI measurement reduced from 17.3 nm to 11.4 nm (with standard deviation σ = 2.3 nm), compared with the results of the offline measurement of 8.7 nm (with standard deviation σ = 1.2 nm). After compensation, the averaged flatness characterization accuracy from the DRI on-machine measurement was improved by 67%. It is noted that the offline measurement needs to be aligned to perform the comparison and the alignment process would inevitably result in some deviation between the two measurements, as shown in Figure 17. 

## 6. Conclusions

This paper presents kinematics error modelling, measurement, and compensation for on-machine surface measurement. Both theoretical and experimental work has been conducted to generate the machine tool kinematics error map for compensation of the OMSM results. Conclusions of the present work can be summarized as follows:

(1) A selective kinematics error model for a three-axis ultra-precision turning machine was established, based on multi-body theory and homogeneous transformation matrix (HTM). Selected error components *E_ZX_*, *E_ZC_*, *E_BC_*, and *E_BOC_* in the OMSM sensitive direction were measured using the reversal method. From the generated error map, it can be seen that the machine tool kinematics error was dominated by the *C* axis tilt error *E_BC_* with a 2 upr component.
(2) The OMSM experiment of an optically flat mirror has shown the DRI measurement result comprises the sample surface form error and the machine tool kinematics error. After the proposed kinematics error compensation, the flatness characterization accuracy from the DRI on-machine measurement was improved by 67%.

## Figures and Tables

**Table 1:** Kinematics transformation matrices between adjacent coordinates.

Symbol	Kinematics Transformation Matrices Between Adjacent Coordinates
T10	$\left[\begin{array}{cccc} 1 & 0 & 0 & p_{1x}\\ 0 & 1 & 0 & p_{1y}\\ 0 & 0 & 1 & p_{1z}\\ 0 & 0 & 0 & 1 \end{array}\right]\left[\begin{array}{cccc} 1 & 0 & 0 & 0\\ 0 & 1 & 0 & 0\\ 0 & 0 & 1 & 0\\ 0 & 0 & 0 & 1 \end{array}\right]\left[\begin{array}{cccc} 1 & 0 & 0 & x\\ 0 & 1 & 0 & 0\\ 0 & 0 & 1 & 0\\ 0 & 0 & 0 & 1 \end{array}\right]\left[\begin{array}{cccc} 1 & -E_{CX} & E_{BX} & E_{XX}\\ E_{CX} & 1 & -E_{AX} & E_{YX}\\ -E_{BX} & E_{AX} & 1 & E_{ZX}\\ 0 & 0 & 0 & 1 \end{array}\right]$
T21	$\left[\begin{array}{cccc} 1 & 0 & 0 & p_{2x}\\ 0 & 1 & 0 & p_{2y}\\ 0 & 0 & 1 & p_{2z}\\ 0 & 0 & 0 & 1 \end{array}\right]\left[\begin{array}{cccc} 1 & 0 & E_{BOC} & 0\\ 0 & 1 & -E_{AOC} & 0\\ -E_{BOC} & E_{AOC} & 1 & 0\\ 0 & 0 & 0 & 1 \end{array}\right]\left[\begin{array}{cccc} \cos\left(\theta \right) & -\sin\left(\theta \right) & 0 & 0\\ \sin\left(\theta \right) & \cos\left(\theta \right) & 0 & 0\\ 0 & 0 & 1 & 0\\ 0 & 0 & 0 & 1 \end{array}\right]\;\left[\begin{array}{cccc} 1 & -E_{CC} & E_{BC} & E_{XC}\\ E_{CC} & 1 & -E_{AC} & E_{YC}\\ -E_{BC} & E_{AC} & 1 & E_{ZC}\\ 0 & 0 & 0 & 1 \end{array}\right]$
T32	$\left[\begin{array}{cccc} 1 & 0 & 0 & p_{3x}\\ 0 & 1 & 0 & p_{3y}\\ 0 & 0 & 1 & p_{3z}\\ 0 & 0 & 0 & 1 \end{array}\right]$
T40	$\left[\begin{array}{cccc} 1 & 0 & 0 & p_{4x}\\ 0 & 1 & 0 & p_{4y}\\ 0 & 0 & 1 & p_{4z}\\ 0 & 0 & 0 & 1 \end{array}\right]\left[\begin{array}{cccc} 1 & 0 & E_{BOZ} & 0\\ 0 & 1 & 0 & 0\\ -E_{BOZ} & 0 & 1 & 0\\ 0 & 0 & 0 & 1 \end{array}\right]\left[\begin{array}{cccc} 1 & 0 & 0 & 0\\ 0 & 1 & 0 & 0\\ 0 & 0 & 1 & z\\ 0 & 0 & 0 & 1 \end{array}\right]\left[\begin{array}{cccc} 1 & -E_{CZ} & E_{BZ} & E_{XZ}\\ E_{CZ} & 1 & -E_{AZ} & E_{YZ}\\ -E_{BZ} & E_{AZ} & 1 & E_{ZZ}\\ 0 & 0 & 0 & 1 \end{array}\right]$
T54	$\left[\begin{array}{cccc} 1 & 0 & 0 & p_{5x}\\ 0 & 1 & 0 & p_{5y}\\ 0 & 0 & 1 & p_{5z}\\ 0 & 0 & 0 & 1 \end{array}\right]$

**Figure A1:**
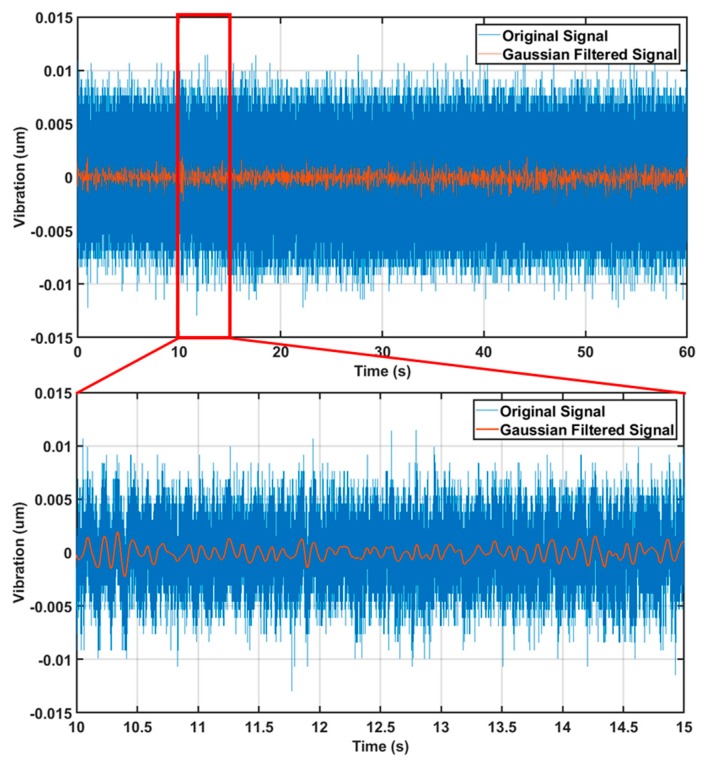
Static test of the capacitance probe.

## Appendix A

In order to check the drift issue, the testing of the used capacitance probe is described in this section. In the experiment, the capacitance probe was mounted on the machine tool Z axis and measured the mirror at a fixed position, as shown in Figure 13. The duration of the measurement was 60 s. The high-frequency electronic noise was filtered out using a Gaussian low-pass filter (60 Hz). After low-pass filtration, the noise level can be as low as 0.6 nm (root mean square). The result shown in Figure A1 does not indicate the drift of the used capacitance probe.
